# Reporting Extended-Spectrum β-Lactamase Positivity May Reduce Carbapenem Overuse

**DOI:** 10.1093/ofid/ofz064

**Published:** 2019-02-19

**Authors:** Pranita D Tamma, Sara E Cosgrove, Edina Avdic, Karen C Carroll, Patricia J Simner

**Affiliations:** 1 Department of Pediatrics, Johns Hopkins University School of Medicine, Baltimore, Maryland; 2 Department of Medicine, Johns Hopkins University School of Medicine, Baltimore, Maryland; 3 Department of Pharmacy, Johns Hopkins University School of Medicine, Baltimore, Maryland; 4 Department of Pathology, Johns Hopkins University School of Medicine, Baltimore, Maryland

To the Editor—In light of the results of the first randomized clinical trial indicating poorer outcomes for patients with presumed extended-spectrum β-lactamase (ESBL) bloodstream infections treated with piperacillin-tazobactam compared with carbapenem therapy [1], there has been a renewed interest in understanding the role of confirmatory ESBL testing. In 2010, the Clinical Laboratory and Standards Institute lowered the ceftriaxone breakpoint from 8 to 1 μg/mL and removed the endorsement for routine confirmatory ESBL testing [2].

Available investigations identifying the ceftriaxone minimum inhibitory concentration (MIC) most predictive of ESBL production indicate that although ESBL producers are likely to have ceftriaxone MICs that are not susceptible, not all Enterobacteriaceae with ceftriaxone MICs that test not susceptible are ESBL producers [3–5]. Because many microbiology laboratories have abandoned ESBL testing, this may result in clinicians using ceftriaxone MICs ≥2 μg/mL as a proxy for ESBL production, resulting in the prescription of carbapenems rather than other susceptible antibiotics (eg, cefepime and piperacillin-tazobactam), even for Enterobacteriaceae not producing ESBLs.

Our objective was to determine the impact ESBL reporting might have on carbapenem use. We hypothesized that ESBL reporting may lead to a reduction in carbapenem use because it takes the “guesswork” out of presuming that Enterobacteriaceae isolates with ceftriaxone MICs ≥2 μg/mL are always likely to produce ESBLs. We compared the proportion of patients anticipated to receive carbapenem therapy if ESBL confirmatory testing had been reported to clinicians with the proportion prescribed carbapenem therapy with ceftriaxone MICs ≥2 μg/mL for these same organisms, but without reporting ESBL status to prescribers for the latter group.

We used ESBL ETESTs (bioMérieux) to identify ESBL production based on ceftriaxone and/or ceftazidime MIC ≥2 μg/mL in *Escherichia coli*, *Klebsiella pneumoniae*, *Klebsiella oxytoca*, and *Proteus mirabilis* clinical isolates collected from 668 unique patients over a 6-week time period at Johns Hopkins Hospital. Any isolates resistant to carbapenem antibiotics were excluded from further analysis. The overall ceftriaxone MIC distribution of the 668 unique isolates were as follows: ≤1 μg/mL, 488 isolates (73%); 2 μg/mL, 24 isolates (4%); 4 μg/mL, 21 isolates (3%); 8 μg/mL, 67 isolates (10%); and ≥16 μg/mL, 68 isolates (10%).

ESBL positivity was identified in 18% of unique patient isolates (118 of 668) for these same species during this period. Of isolates with ceftriaxone MICs ≥2 μg/mL, 66% (118 of 180) were ESBL producers. The distribution of ESBL-positive organisms was as follows: *E. coli,* 59%; *K. pneumoniae,* 27%; *K. oxytoca,* 5%; and *P. mirabilis,* 9%.

Additional testing was performed on 5 isolates identified as indeterminate by the ETEST using the Check-MDR CT101 Microarray (Check-Points), with 4 isolates containing both ESBL and AmpC genes and 1 isolate containing both an ESBL and carbapenemase gene. Specific β-lactamase genes identified in the 5 indeterminate isolates included (1) *bla*_CTX-M-9-group_, *bla*_DHA_ (*E. coli*); (2) *bla*_CTX-M-9-group_, *bla*_DHA_ (*E. coli*); (3) *bla*_SHV-type_, *bla*_CTX-M-1-group_, *bla*_FOX_ (*K. pneumoniae*); (4) *bla*_CTX-M-9 group_, *bla*_DHA_ (*E. coli*); and (5) *bla*_SHV-type_, *bla*_KPC_ (*K. pneumoniae*).

The median ceftriaxone MIC for ESBL-producing isolates was 16 μg/mL (interquartile range, 16–32 μg/mL). Of 118 ESBL-positive isolates, 3 (3%) had ceftriaxone MICs of 2 μg/mL and 2 (2%) had ceftriaxone MICs of 8 μg/mL, with all other ESBL-positive ceftriaxone MICs ≥16 μg/mL (113 of 118; 95%). There were 107 isolates with ceftriaxone MICs between 2 and 8 μg/mL that were ESBL negative by ETEST.

Of patients infected with *E. coli*, *Klebsiella* spp., or *P. mirabilis* with ceftriaxone MICs ≥2 μg/mL (in the absence of reporting ESBL testing results to clinicians), excluding those already receiving empiric carbapenem therapy and those with isolates not susceptible to either cefepime or piperacillin-tazobactam, 62% were transitioned to carbapenem therapy after ceftriaxone MIC data were available. If ESBL results were reported to clinicians (with indeterminate results inferred to be positive), we anticipate that 21% of patients would be transitioned to carbapenem therapy, again excluding patients already receiving carbapenem therapy and those without susceptibility to cefepime and piperacillin-tazobactam (*P* <.01). With the ongoing gram-negative resistance crisis, our results suggest that it could be helpful to refine the process of ESBL detection and evaluate, on a larger scale, whether confirmatory ESBL testing could reduce carbapenem overuse.
